# Gait analysis in older adults with mild cognitive impairment: a bibliometric analysis of global trends, hotspots, and emerging frontiers

**DOI:** 10.3389/fragi.2025.1592464

**Published:** 2025-06-19

**Authors:** Yuan Zhong, Siqi Huang, Meixia Zou, Yiming Chen, Peifeng Shen, Yanan He, Yuanchao Li, Chunlong Liu, Zhibiao Chen

**Affiliations:** ^1^ Zhongshan Hospital of Traditional Chinese Medicine, Zhongshan, China; ^2^ Clinical Medical College of Acupuncture Moxibustion and Rehabilitation, Guangzhou University of Chinese Medicine, Guangzhou, China

**Keywords:** gait analysis, mild cognitive impairment, bibliometric analysis, research hotspots, dementia

## Abstract

**Background:**

Gait analysis has emerged as a critical non-invasive tool for early identification and monitoring of mild cognitive impairment (MCI) in aging populations, particularly given its potential to predict dementia progression. This bibliometric analysis synthesizes two decades of research to map the evolution of gait analysis in MCI, identify interdisciplinary collaborations, and highlight emerging frontiers in MCI-related mobility research.

**Methods:**

Literature related to gait analysis in MCI was retrieved from the Web of Science Core Collection. The search spanned publications from 2005 to 2024 and was executed in a single search session on 15 December 2024. CiteSpace and VOSviewer software were used to analyze publications, authorship, institutional affiliations, journals, keywords, and cited references. Burst detection and timeline analyses of keywords and references were conducted to identify emerging trends and temporal patterns.

**Results:**

A total of 1,223 articles were identified. Annual publication trends indicate sustained scholarly interest over the past 5 years. The United States contributed the most publications (392 articles, 32.05%), with Western University (Canada, 65 articles) as the leading institution. Journals publishing these studies primarily focus on Alzheimer’s disease (AD), gerontology, and neurology, while prolific authors like Verghese J (USA) and Montero-odasso M(Canada) shaped the field’s trajectory. Emerging research frontiers include dementia progression, AD, and Parkinson’s disease, with 2024 priorities emphasizing “dual-task walking”, “digital biomarkers” and “working groups”. Additionally, validity and reliability assessments of gait analysis for MCI diagnosis and intervention represent a growing research trend.

**Conclusion:**

This study provides a comprehensive overview of the current landscape, hotspots, and trends in gait analysis for MCI management. By delineating its transformation from a descriptive tool to a predictive framework, we highlight persistent challenges such as methodological heterogeneity and small sample sizes. However, advances in machine learning and multicenter collaborations present opportunities to standardize protocols. Future high-quality studies are expected to establish gait-derived biomarkers as clinically actionable tools in MCI stratification and therapeutic monitoring.

## 1 Introduction

Mild cognitive impairment (MCI) is prevalent among older populations, with incidence rates escalating alongside age (Petersen et al., 2014). Characterized by progressive memory or cognitive decline, MCI does not severely impair daily functioning nor meet diagnostic thresholds for dementia (Jia et al., 2020). Epidemiological studies indicate that older adults with MCI face a significantly elevated risk of progressing to dementia, particularly Alzheimer’s disease (AD), with an estimated 10%–15% of MCI patients transitioning to AD annually (Nelson et al., 2021; Author Anonymous, 2024).

As an intermediate transitional stage between normal aging and dementia (Anderson, 2019), MCI exhibits variable trajectories: cognitive decline may stabilize, reverse, or deteriorate into dementia (Petersen, 2004; Soldan et al., 2017). Consequently, early detection and intervention in older adults experiencing cognitive decline present a critical opportunity to delay progression from MCI to dementia. Such efforts are vital for mitigating disease severity and enhancing patients’ quality of life (Langa and Levine, 2014).

Although MCI primarily manifests as cognitive decline, the deterioration of cognitive function (including executive function and visuospatial structure function) is frequently accompanied by subtle gait abnormalities (Li et al., 2022). Cognitive decline manifests as deterioration in memory, attention, and executive function, while gait abnormalities include slow gait speed, gait initiation failure, and increased fall risk, all of which subtly impact daily functioning (Allali et al., 2017; Lindh-Rengifo et al., 2022; Kim et al., 2023). In 2003, a multidisciplinary and international expert panel (comprising global specialists from Asia, Australia, Europe, and North America) explicitly concluded through structured discussions that integrating gait analysis and neuropsychological testing could aid in both early detection of MCI and prediction of its clinical progression, reaching a consensus (Winblad et al., 2004). According to Chinese clinical guidelines (evidence of level I) (CDYcDdatgc, 2018), gait performance serves as a biomarker for identifying cognitive decline. Gait analysis technology leverages anatomical and physiological principles to assess walking patterns and quantify spatiotemporal and joint kinematics parameters. These metrics help detect gait alterations linked to cognitive decline, thereby identifying potential biomarkers for MCI (Zhong et al., 2021; Cicirelli et al., 2022; Aznielle-Rodríguez et al., 2023). Aging involves biomolecular mechanisms such as cellular senescence, mitochondrial dysfunction, and epigenetic alterations, which disrupt processes like genomic stability and proteostasis, ultimately impairing cognitive and motor functions (Wagner et al., 2016; Rizzuto et al., 2017). Thus, integrating gait analysis with cognitive assessments in clinical and research settings highlights the critical interplay between physiological and neurological health.

Although gait studies in MCI have expanded significantly, existing reviews primarily synthesize foundational literature (Doi et al., 2017; Pieruccini-Faria et al., 2020; Zhong et al., 2021), with limited focus on mapping research trends, hotspots, or developmental trajectories. Bibliometric analysis, a quantitative statistical method, examines patterns in academic research to evaluate its characteristics and societal impact, and has been widely applied across disciplines (Hicks et al., 2015). Despite advances in MCI-related gait research, the absence of bibliometric analyses hinders comprehensive synthesis of progress, posing challenges for researchers.

To address this gap, we employed Microsoft Excel, CiteSpace and VOSviewer to visually analysis of the publication, countries, institutions, journals, authors, keywords and references in MCI gait studies over the past 20 years. We have conducted a comprehensive analysis of literature spanning 2005 to 2024 in this field, with particular focus on delineating the current research landscape, emerging frontiers, and global hotspots and trends. This bibliometric Analysis highlights the growing clinical significance of gait-MCI correlations while identifying critical barriers to its broader acceptance, ultimately offering actionable insights for future investigations by researchers and clinical practitioners.

## 2 Methods and material

### 2.1 Data sources and search strategies

The Web of Science (WoS) is a globally influential and authoritative comprehensive literature database. For this bibliometric analysis, data were retrieved exclusively from the Web of Science Core Collection (WoSCC), specifically the Science Citation Index Expanded (SCI-EXPANDED), which covers multidisciplinary scientific fields such as life sciences, health sciences, and physical sciences (Zhang et al., 2024).

Based on the relevance of topic to the study’s focus on gait analysis to identify older adults with MCI, the literature search strategy was (((TS= (gait* OR “gait analysis” OR “gait analyses” OR “walk*”) AND TS= (MCI OR “mild cognitive impairment*” OR “mild cognitive dysfunction*” OR “mild cognitive disorder*” OR “mild cognitive disability” OR “mild neurocognitive disorder” OR “mild vascular cognitive disorder”)) AND TS= (older adults OR “Aged” OR “elder* people” OR “old* people” OR “senior citizen*” OR “old folk*”))).

**FIGURE 1:**
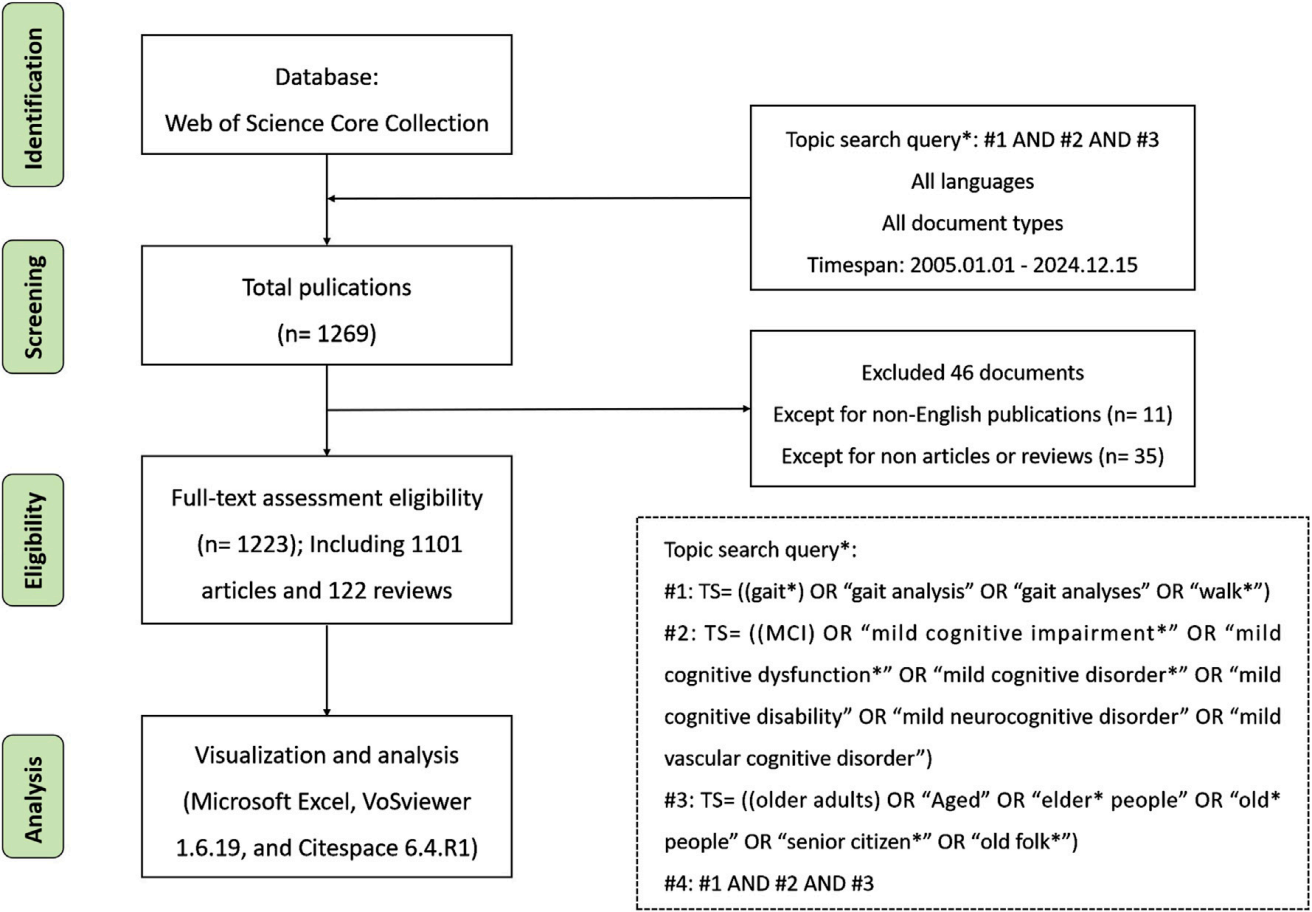
Flowchart of screening process.

The search was conducted on 15 December 2024, and restricted to publications from 2005 to 2024. Inclusion criteria: available in the English language, and only articles and reviews were included. Exclusion criteria: publications in languages other than English, studies conducted outside the specified time frame, and other types of documents, such as conference papers, books, and letters, were excluded. Ultimately, articles, including prospective studies and reviews, were retrieved and subjected to analysis. After screening, 1,269 records were retrieved, of which 46 were deemed irrelevant. The final 1,223 articles were exported as plain text files (full records and references) for advanced analysis. The search workflow is illustrated in Figure 1.

### 2.2 Bibliometric analysis and visualization

The outcomes of bibliometric analysis encompass performance metrics such as contributions by countries, institutions, journals, authors, keywords, and references (Song et al., 2022). To ensure comprehensive insights into gait analysis and MCI in older adults populations, this study employed a multi-tool approach: Microsoft Office Excel for data management and trend analysis, CiteSpace for visualizing research networks and frontiers, and VOSviewer for constructing bibliometric networks.

Citespace is a widely useful bibliometric visualization analysis software, mainly used to describe research hotspots, explore existing scientific fields and academic networks, and identify research trends (Chen, 2004; Chen, 2006). CiteSpace generates a network map comprising tree-ring nodes and connecting lines. The tree-ring nodes denote analyzed entities (e.g., keywords, institutions), with node size reflecting frequency, color indicating publication year, and purple peripheries signaling high betweenness centrality (critical nodes in collaboration networks) (Liu et al., 2019). The connecting lines represent co-occurrence relationships, with thickness denoting connection strength and color marking the year of first co-occurrence. Cool-to-warm color transitions reflect temporal progression (Song et al., 2022). Additionally, the timeline viewer facilitates historical cluster analysis, identifies foundational references within clusters, and tracks fluctuations in scholarly attention over time. Occurrence bursts in CiteSpace, characterized by abrupt increases in term frequency during defined time intervals, serve as indicators of paradigm shifts in research frontiers and disciplinary dynamics (Ma et al., 2021; Xu et al., 2024). Nodes exhibiting active burst periods are projected to maintain this growth trajectory for a foreseeable duration (Chen et al., 2024). Furthermore, CiteSpace’s dual-map overlay function visualizes knowledge transfer patterns: the left matrix maps citing journals (reflecting emerging research domains), while the right matrix positions cited journals (denoting foundational literature). The curvature bandwidth quantitatively represents citation intensity through two metrics—fluctuation magnitude (z-score) and occurrence density (f-score) (Yang et al., 2024).

VOSviewer is a non-commercial tool based on VOS technology, specializes in visualizing bibliometric networks through co-occurrence, citation, and co-citation relationships (Fu et al., 2022). In a collaboration network, each node represents a different object, and the size of the nodes is determined by their frequency of occurrence and citation, or the weight of their connections (Arruda et al., 2022). The connecting lines between nodes represent their degree of contact, and the thicker lines indicate stronger links. Various clusters are represented in different colors and often represent different directions of research (Sun et al., 2024).

This study employed Microsoft Excel 2019 to analyze publication trends over the past two decades and forecast future publication volumes, while also facilitating data management (Liu et al., 2024). CiteSpace 6.4. R1 generated collaborative network graphs for countries, institutions, and dual-map overlays of journals, alongside identifying citation bursts for keywords and references. Centrality metrics were applied to identify and quantify influential nodes within these networks (Jiao et al., 2022). By detecting citation bursts in keywords and references, emerging research frontiers were identified, reflecting concentrated scholarly attention during specific periods (Chen et al., 2024). VOSviewer 1.6.19 constructed the following networks: co-authorship networks for countries and institutions, citation networks for journals and authors, keyword co-occurrence networks, co-citation networks for authors and references (Sun et al., 2024). In addition, Scimago was used to generate a world map to present the cooperation between countries (Rocamora-Montenegro et al., 2021).

Additionally, in order to reduce human errors during the literature visualization analysis, we adopted a collaborative cross-checking approach involving three researchers for data management and analysis. All bibliometric data collection (WoSCC), management (Excel), and analysis (CiteSapce and VOSviewer) in this study were conducted through a collaborative cross-checking system involving three researchers to minimize human error. The research process encompassing literature retrieval, citation data importation into visualization software for analysis, and subsequent data management that were systematically executed using two independent computer systems to ensure consistency across all operational phases. Any identified discrepancies were resolved through iterative verification by a third researcher until complete analytical consensus was achieved among all three investigators.

## 3 Results

### 3.1 Annual publications and trends

**FIGURE 2:**
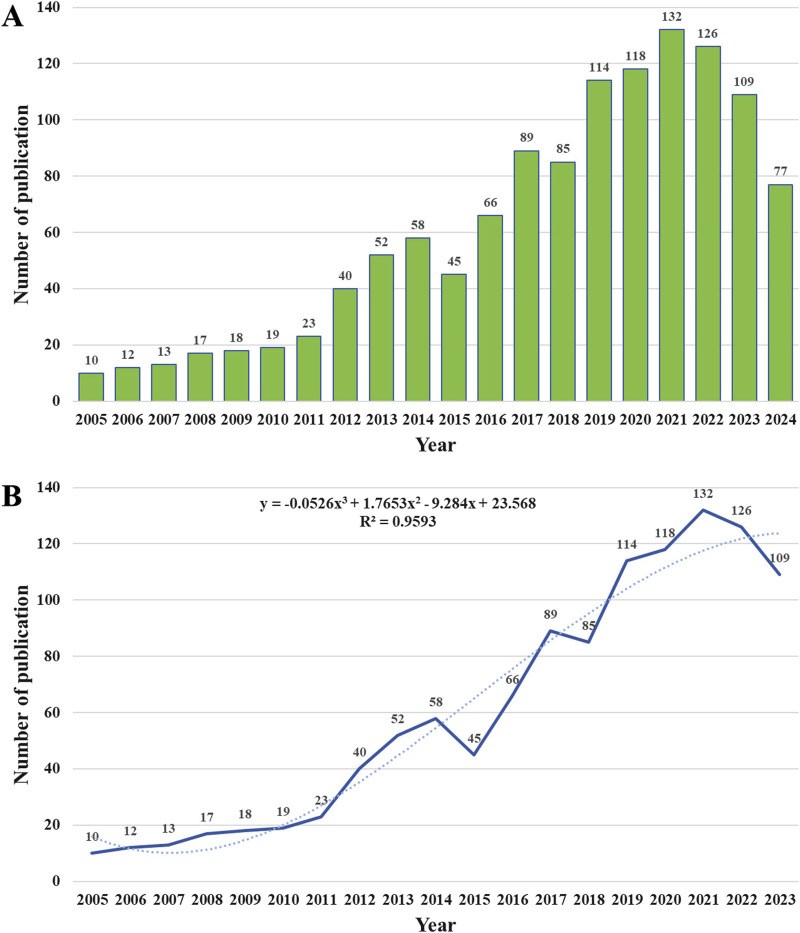
Publication outputs and time trend of gait studies on MCI. **(A)** The number of annual publications from 2005 to 2024. **(B)** The model fitting curve of time trend of publications.

The deduplication process, performed using CiteSpace, confirmed the absence of duplicate records, resulting in a final inclusion of 1,223 publications. Figure 2A illustrates annual publication counts from 2005 to 2024, while Figure 2B presents a polynomial regression model (R^2^ = 0.9593) fitted to cumulative publication data, indicating a transition toward a plateau phase in MCI-related gait research. Publications surged from 10 in 2005 to 132 in 2021, peaking before stabilizing at 109 in 2023. This fluctuating yet upward trajectory underscores sustained scholarly focus on gait analysis in older adults with MCI, particularly over the past 5 years. The retrieval was conducted on 15 December 2024, and the search showed that 77 studies were published that year. Accounting for publication delays and ongoing research advancements, further growth in novel gait studies targeting MCI is anticipated.

### 3.2 Analysis of countries/regions and institutions

Over the past 20 years, a total of 70 countries and 2,067 institutions contributed 1,223 publications on gait analysis in MCI. Table 1 summarizes the top 10 countries and institutions by publication volume and centrality. The United States led with 392 publications (32.05%), followed by Canada (145), China (134), Japan (116), and England (90). Among institutions, Western University ranked first (65 publications, 5.31%), followed by Albert Einstein College of Medicine and Montefiore Medical Center (48 each). Notably, 60% of the top 10 institutions were U.S.-based.

**TABLE 1:** Top 10 most productive countries/regions and institutions.

Rank	Country	Count (%)	Centrality	Institution (Country)	Count (%)	Centrality
1	United States	392 (32.05%)	0.25	Western University (Canada)	65 (5.31%)	0.10
2	Canada	145 (11.86%)	0.04	Albert Einstein College of Medicine (United States)	48 (3.92%)	0.04
3	China	134 (10.96%)	0.09	Montefiore Medical Center (United States)	48 (3.92%)	0.03
4	Japan	116 (9.48%)	0.02	Yeshiva University (United States)	48 (3.92%)	0.02
5	England	90 (73.59%)	0.25	National Center for Geriatrics & Gerontology (Japan)	43 (3.52%)	0.06
6	Australia	84 (6.87%)	0.15	Pennsylvania Commonwealth System of Higher Education (United States)	33 (2.70%)	0.22
7	Italy	79 (6.46%)	0.08	Harvard University (United States)	31 (2.53%)	0.07
8	France	73 (5.97%)	0.03	University of California System (United States)	30 (2.45%)	0.08
9	Germany	62 (5.07%)	0.12	Tel Aviv University (Israel)	26 (2.13%)	0.08
10	Spain	60 (4.91%)	0.06	MiGill University (Canada)	26 (2.13%)	0.02

**FIGURE 3:**
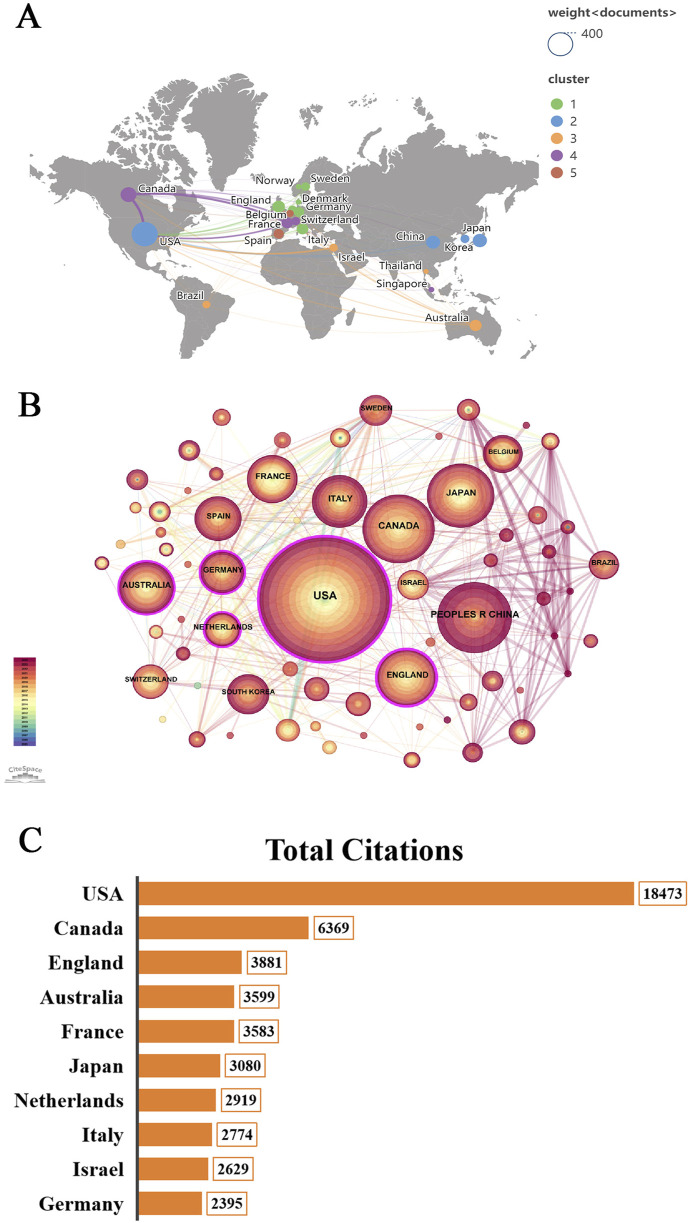
The co-authorship analysis of the countries or regions. **(A)** Map of world distribution of the publications within this domain. **(B)** Collaborations map of countries or regions. **(C)** The top ten countries with the highest aggregate citation count.

Figure 3A illustrates the international collaboration network, where node size corresponds to countries with ≥15 publications (21 total). Line thickness reflects collaboration strength. The U.S. dominated with a total link strength of 219, followed by Canada (152) and England (144). Figure 3B maps MCI gait research across 70 countries/regions, with node size indicating publication volume, purple circles denoting centrality (higher values = greater influence), and tree-ring colors (purple to red) signaling publication recency. The U.S. exhibited the highest centrality (0.25), alongside England (0.25), Australia (0.15), Germany (0.12), and the Netherlands (0.12). Canada and China, though low in centrality, showed recent activity (past 5 years) and collaborations with emerging nations (e.g., South Korea, Sweden, Brazil). Figure 3C highlights citation dominance by the U.S. (18,473 citations), followed by Canada, England, Australia, and France (all >3,500).

**FIGURE 4:**
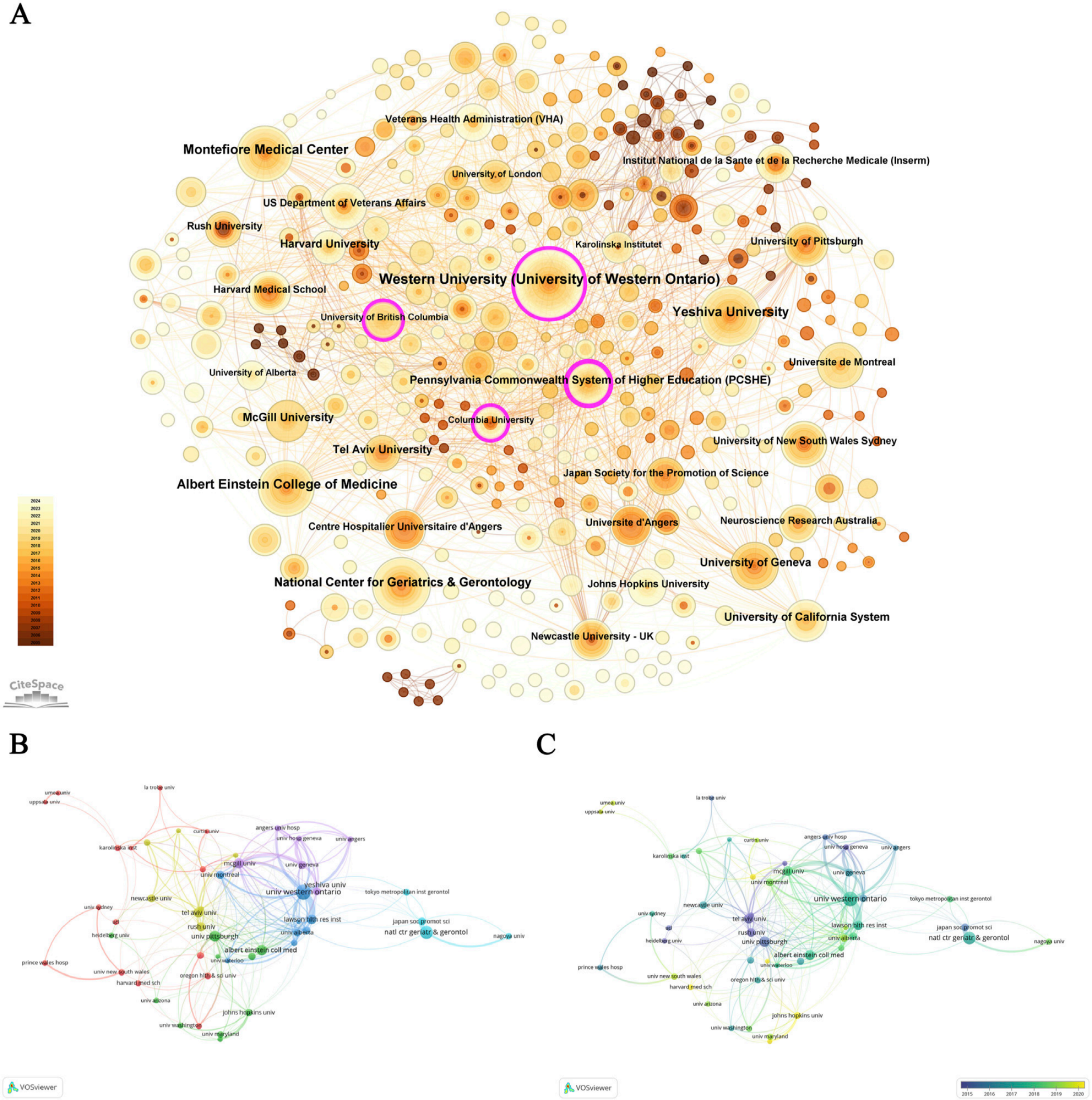
The co-authorship analysis of the institutions. **(A)** Collaborations map of institutions. **(B)** Network visualization portraying the co-authorship interconnections among institutions with more than 10 publication. **(C)** Network visualization illustrating the co-authorship interconnections among institutions, categorized based on the mean publication year (blue denoting earlier years, and yellow denoting later years).

Institutional collaboration (Figure 4A) revealed Western University as the most prolific (65 publications, centrality = 0.10), while the Pennsylvania Commonwealth System of Higher Education (U.S.) had the highest centrality (0.22). Figure 4B visualizes a network of 56 institutions (≥10 publications each), clustered by color. Western University (Canada) led in link strength (126), followed by McGill University (92). Most influential nodes (e.g., Albert Einstein College of Medicine, University of Pittsburgh) were U.S.-based. Figure 4C indicates that average publication years for these institutions postdate 2015, underscoring intensified research focus over the past decade.

### 3.3 Analysis of journals

Since 2005, articles on gait studies in MCI have been published across 333 journals. Table 2 enshrines the top 10 journals distinguished by the highest publication. Among these journals, the Journal of Alzheimer’s Disease led with 47 publications, followed by Frontiers in Aging Neuroscience (46) and the Journals of Gerontology Series A: Biological Sciences and Medical Sciences (43). Notably, the Journal of the American Geriatrics Society achieved the highest impact factor (IF = 7.5) and citation count (2,995 citations).

**TABLE 2:** Top 10 academic journals based on publications.

Rank	Top journals	Publications	Citations	If (2023)^a^	JCR
1	Journal of Alzheimer’s Disease	47	1,060	3.4	Q2
2	Frontiers in Aging Neuroscience	46	1,022	4.1	Q2
3	Journals of Gerontology Series A-biological Sciences and Medical Sciences	43	2,340	4.3	Q1
4	BMC Geriatrics	40	734	3.4	Q2
5	PLoS One	34	1,270	2.9	Q1
6	Journal of the American Geriatrics Society	28	2,995	7.5	Q1
7	Experimental Gerontology	26	619	3.3	Q2
8	Gait & Posture	22	856	2.2	Q2
9	Journal of the American Medical Directors Association	19	925	4.2	Q2
10	Aging Clinical and Experimental Research	19	531	3.4	Q2

^a^ IF according to Journal Citation Reports (2023).

Abbreviations: IF, impact factor; JCR, journal citation reports, Q = quartile in category.

**FIGURE 5:**
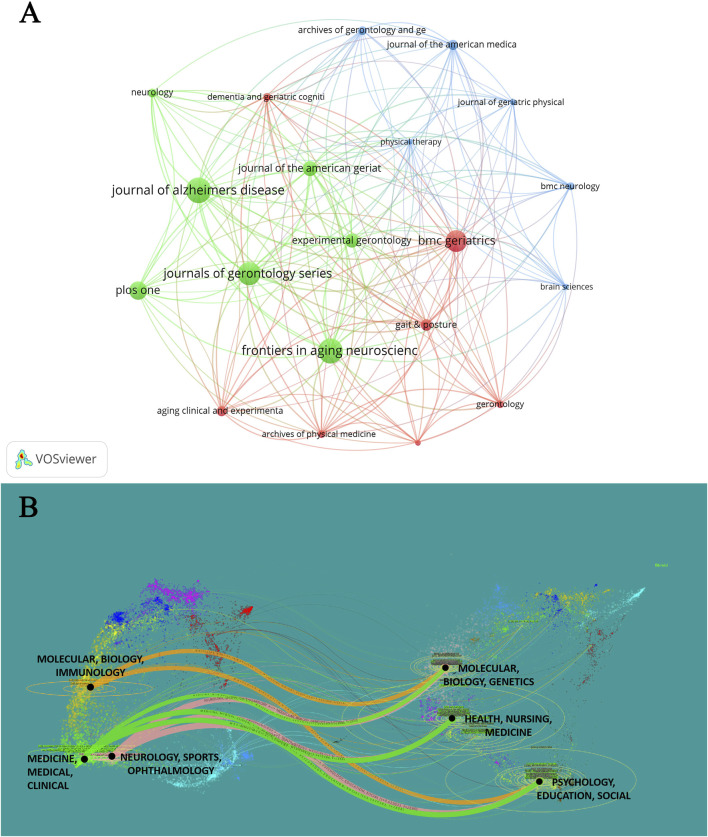
Analysis of journals. **(A)** Distribution from different journals of gait studies on MCI. **(B)** The dual-map overlay of journals with publications.

A collaborative network analysis of 28 journals with ≥10 publications in this field revealed three distinct clusters (Figure 5A): the green cluster comprised geriatric and cognitive neurology journals; the red cluster focused on clinical gait analysis and geriatric physiotherapy; and the blue cluster emphasized brain science and mechanistic or therapeutic research in geriatric mobility. Employing a dual-map overlay (Figure 5B), we traced citation pathways between citing and cited journals in different subject categories. Three key pathways—highlighted in bright green, pink, and orange—demonstrated interdisciplinary linkages. The pink pathway having the highest citation intensity, which indicated current subjects neurology, sports, and ophthalmology mainly reference to the basic subject research, including molecular, biology, and genetics (z = 4.62, f = 2,290), health, nursing, and medicine (z = 2.91, f = 1,514) and psychology, education, and social (z = 3.92, f = 1974). Furthermore, Figure 5B further illustrates that MCI-related gait research bridges emerging fields (e.g., clinical medicine, neurology) with foundational disciplines such as molecular biology, psychology, and social sciences.

### 3.4 Analysis of authors and co-cited authors

**FIGURE 6:**
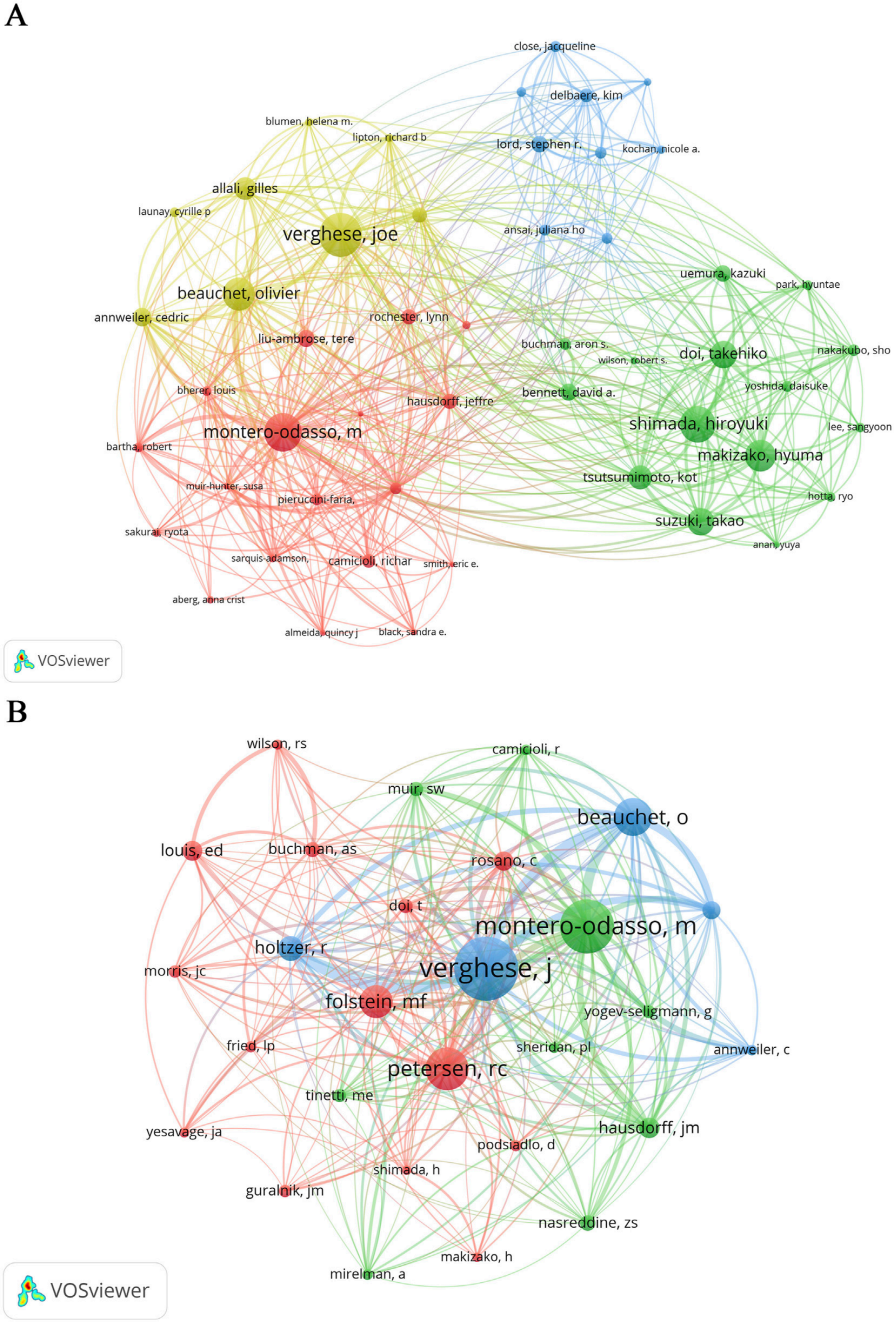
Analysis of active authors. **(A)** Network visualization highlighting the co-authorship connections among top 50 authors who have contributed to more than five publications. **(B)** Network visualization illustrating the co-cited authors who have received over 100 citations.

A total of 6,554 authors contributed to the analyzed publications. Co-cited authors are defined as two or more researchers cited together in subsequent papers, enabling the identification of scholars with closely aligned research collaborative interests. Table 3 highlights ten authors distinguished by their publication volume, citation counts, and centrality scores. Verghese J (Albert Einstein College of Medicine) was the most prolific, with 40 publications, followed by Montero-odasso M (Western University; 35 publications) and Shimada H (Stanford University Medical Center; 33 publications). Verghese J also led in citations (696), ahead of Montero-odasso M (587). Tinetti ME recorded the highest centrality score (0.14), with Verghese ranking second (0.11). We systematically identified the top 50 authors who have collaboratively contributed to a substantial number of publications ranging from 5 to 40 (Figure 6A). Manuel Montero-odasso M, Verghese J, and Speechley M demonstrated the highest total link strength (49, 49, and 48, respectively), reflecting their dominant collaborative influence. Figure 6B maps co-citation relationships among 28 authors with ≥100 citations, revealing a tightly interconnected network. The total link strength (26–27) suggests pervasive collaborative relationships, indicating that nearly all author pairs share significant academic symbiosis (Xu et al., 2024).

**TABLE 3:** Top 10 most active authors and co-cited authors.

Rank	Author	Country	Number of publications	Co-cited authors	Cite times	Co-cited authors	Centrality
1	Verghese J	United States	40	Verghese J	696	Tinetti ME	0.14
2	Montero-odasso M	Canada	35	Montero-odasso M	587	Verghese J	0.11
3	Shimada H	Japan	33	Petersen RC	466	Petersen RC	0.09
4	Beauchet O	Canada	30	Beauchet O	413	Rosano C	0.09
5	Makizako H	Japan	29	Folstein MF	357	Holtzer R	0.08
6	Doi T	Japan	25	Holtzer R	273	Yesavage JA	0.07
7	Suzuki T	Japan	25	Hausdorff JM	224	Camicioli R	0.07
8	Tsutsumimoto K	Japan	22	Rosano C	208	Folstein MF	0.06
9	Annweiler C	France	17	Allali G	199	Muir SW	0.06
10	Speechley M	England	11	Muir SW	155	Montero-odasso M	0.06

### 3.5 Analysis of keywords

**FIGURE 7:**
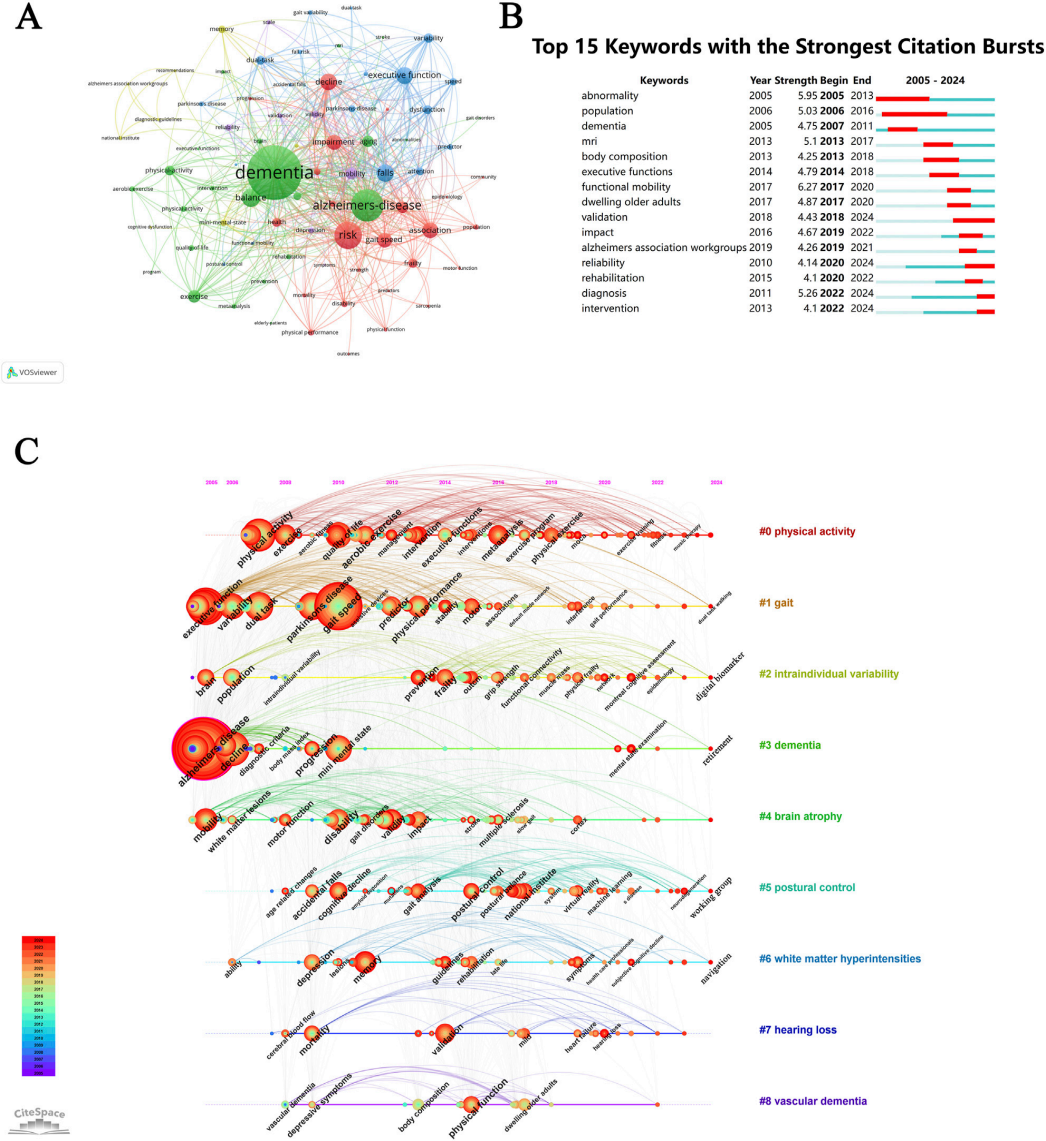
The co-occurrence analysis of the keywords. **(A)** Collaborations map of keywords. **(B)** Keywords with the strongest occurrence burst. **(C)** Timeline map of keywords.

According to Table 4, the most frequent keyword in this study over the past two decades is “Alzheimer’s disease”, followed by “dementia”, both of which also exhibit high centrality. As shown in Figure 7A, we can see the clusters of green, red, blue and yellow, which respectively represent four distinct research directions.

**TABLE 4:** Top 10 keywords with the highest frequency and centrality.

Rank	Keywords	Frequency	Keywords	Centrality
1	Alzheimers disease	441	dementia	0.13
2	dementia	381	Alzheimers disease	0.10
3	risk	332	Parkinsons disease	0.07
4	gait speed	238	aerobic exercise	0.07
5	executive function	154	gait variability	0.07
6	decline	152	decline	0.06
7	falls	146	mobility	0.06
8	association	144	attention	0.06
9	physical activity	120	disability	0.06
10	balance	118	brain	0.06

Keywords with occurrence bursts persisting through 2024 signify current research frontiers, where burst strength and duration serve as primary metrics for detection. We detected a total of 15 burst keywords with the help of CiteSpace (Figure 7B). The red bars denote frequent keyword occurrences, while green bars indicate infrequent ones. The red bar spans the burst period (start to end), with strength reflecting its impact magnitude. The top three keywords with the highest strength were “functional mobility” (2017–2020), “abnormality” (2005–2013), and “diagnosis” (2022–2024). The keyword that persisted for the longest duration (10 years) was “population”. The recently observed burst keywords included “validation”, “reliability”, “diagnosis”, and “intervention”.

Figure 7C depicts the timeline viewer for gait studies in MCI, illustrating temporal research hotspots and developmental trends. The figure highlights the top nine clusters, labeled by dominant keywords, with “#0 physical activity” as the largest cluster. Analysis reveals that clusters “#2 intraindividual variability” and “#5 postural control”—mostly displayed in red and positioned on the timeline’s rightmost segment—represent emerging frontiers in recent years. Notable 2024 research priorities include “dual-task walking”, “digital biomarkers”, and “working groups.”

### 3.6 Analysis of references

**FIGURE 8:**
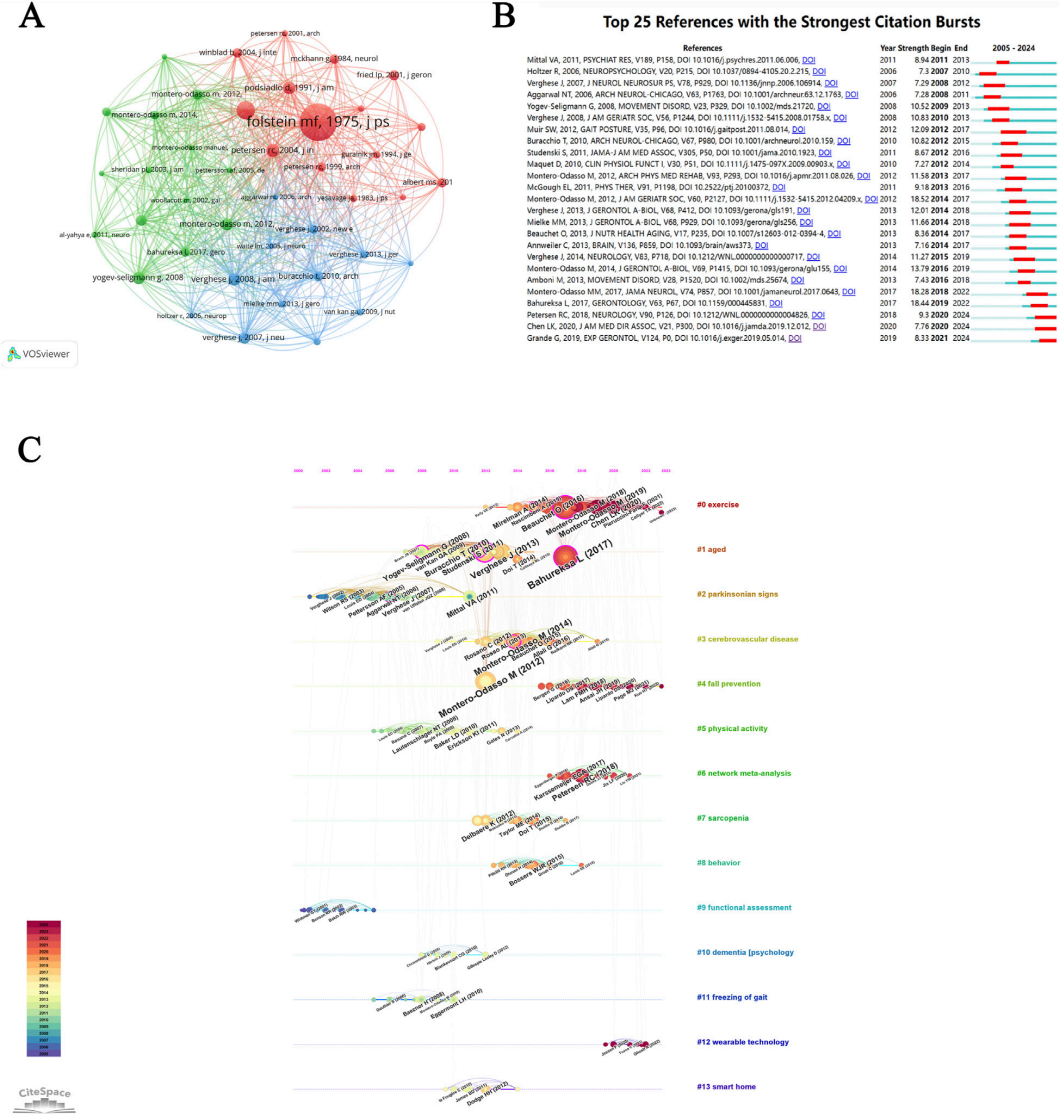
The co-occurrence analysis of the references. **(A)** Collaborations map of co-cited reference. **(B)** References with the strongest citation burst. **(C)** Timeline map of references.

This study analyzed 1,223 publications, yielding 35,646 cited references. Using VOSviewer, we generated a co-citation network of MCI-related gait research over the past two decades (Figure 8A), highlighting 349 highly co-cited articles. Tables 5, 6 list the top five co-cited references and high-centrality gait studies on MCI (contextualized over 20 years), identifying foundational and emerging literature.

**TABLE 5:** Top 5 co-cited references with the highest frequency.

Rank	Title	First author	Year	Frequency
1	The Impact of Mild Cognitive Impairment on Gait and Balance: A Systematic Review and Meta-Analysis of Studies Using Instrumented Assessment	Bahureksa, Lindsay A	2017	59
2	Association of Dual-Task Gait With Incident Dementia in Mild Cognitive Impairment Results From the Gait and Brain Study	Montero-Odasso, Manuel M	2017	56
3	Gait and Cognition: A Complementary Approach to Understanding Brain Function and the Risk of Falling	Montero-Odasso, Manuel M	2012	46
4	Gait assessment in mild cognitive impairment and Alzheimer’s disease: The effect of dual-task challenges across the cognitive spectrum	Muir, Susan W	2012	41
5	The Motor Signature of Mild Cognitive Impairment: Results From the Gait and Brain Study	Montero-Odasso, Manuel M	2014	36

**TABLE 6:** Top 5 co-cited references with the highest centrality.

Rank	Title	First author	Year	Centrality
1	Gait assessment in mild cognitive impairment and Alzheimer’s disease: The effect of dual-task challenges across the cognitive spectrum	Muir, Susan W	2012	0.35
2	Gait dysfunction in mild cognitive impairment syndromes	Verghese, Joe	2008	0.26
3	Neuroimaging of Mobility in Aging: A Targeted Review	Holtzer, Roee	2014	0.17
4	The role of executive function and attention in gait	Yogev-Seligmann, Galit	2017	0.17
5	The Impact of Mild Cognitive Impairment on Gait and Balance: A Systematic Review and Meta-Analysis of Studies Using Instrumented Assessment	Bahureksa, Lindsay A	2017	0.14

Reference bursts signal publications that have undergone sudden surges in attention, reflecting their rising influence. in the field. Figure 8B illustrates the related references with the most pronounced citation bursts over the past 20 years. The 25 articles were arranged in descending order based on the time they gained attention. Among these reference, “Gait and cognition: a complementary approach to understanding brain function and the risk of falling.” (Montero-Odasso et al., 2012b) had the strongest citation burst with 18.52, followed by “The Impact of Mild Cognitive Impairment on Gait and Balance: A Systematic Review and Meta-Analysis of Studies Using Instrumented Assessment.” (Bahureksa et al., 2017) and “Association of Dual-Task Gait With Incident Dementia in Mild Cognitive Impairment: Results From the Gait and Brain Study” (Montero-Odasso et al., 2017).

A timeline analysis (Figure 8C) traces the evolution of MCI gait research through its 14 largest clusters, with the cluster “#0 exercise” being the most prominent, comprising 121 articles. Upon examining the map, we observe that “#4 fall prevention” and “#12 wearable technology” is displayed in red color and positioned towards the right side of the time axis, indicating it as research hotspots in recent years.

## 4 Discussion

This bibliometric analysis examines gait analysis in MCI research from 2005 to 2024, highlighting significant advancements in its application for studying older adults with MCI. We utilized advanced visual management tools, including CiteSpace and VOSviewer, to gain a comprehensive summary of the current status, hotspots, and trends in this field. Gait analysis remains a widely accepted methodology in MCI studies, due not only to its efficacy in identifying early-stage dementia, supporting diagnosis, and guiding intervention strategies, but also to its role in assessing fall risk among older adults and informing the development of portable home-based physical exercise devices.

### 4.1 General information for gait studies on older adults with MCI

The annual publication rate of gait studies on MCI has exhibited fluctuations but overall growth, with significant advancements occurring between 2018 and 2019. Over the past 5 years, publication rates have stabilized, averaging over 100 articles annually, reflecting sustained scholarly interest in MCI-related gait research (Figure 2). This trend may stem from the multifactorial health challenges and clinical complexity of older adults populations with MCI, coupled with its interdisciplinary management framework. Analysis based on the biopsychosocial model of the World Health Organization’s International Classification of Functioning, Disability and Health (WHO-ICF) demonstrates that gait abnormalities in older adults with MCI result from multidimensional interactions within this framework. Within the ICF Body Functions domain, dysfunction in the prefrontal-basal ganglia circuitry may contribute to altered gait cycle phases and increased stride variability (Macfarlane et al., 2015). The Activity domain reflects gait-cognitive dual-task interference induced by executive dysfunction, elevating spatial navigation error rates (Argento et al., 2021). In the Participation domain, reduced community engagement and diminished efficiency in instrumental daily activities among individuals with MCI exhibit a dose-response relationship with impaired gait control (Bracco et al., 2023). Environmental factor analysis in the ICF context indicates that complex terrains substantially heighten fall risk in this population (Pottorf et al., 2022). Increasing recognition of gait abnormalities in MCI by patients and clinicians underscores its future clinical and research significance.

Among the top 10 countries/regions by publication output, the United States leads in productivity, centrality, and citation impact, highlighting robust international collaboration among American researchers (Table 1). England and Australia also exhibit strong centrality, signaling expanding global cooperation in this field. Conversely, Canada and China, despite high publication volumes, currently display lower centrality due to their more recent entry into the field. However, their frequent collaborations with emerging research nations suggest a pioneering role in advancing MCI gait studies (Figure 3).

In terms of institutional contribution, the United States hold a dominant position in the field. Institutions from the United States and Australia played pivotal roles in facilitating collaboration among the research teams (Table 1). In general, most institutions were fragmented, lacking stable and deep cooperation and exchange relationships (Figure 4). While striving to foster innovative discoveries, institutions must further strengthen cooperation with other organizations. By sharing resources, knowledge, and technologies across institutions, global collaboration can accelerate research cycles and optimize clinical solutions. Future efforts should focus on building more efficient collaborative networks to address the increasingly complex health challenges faced by older adults with MCI.

Research on MCI-related gait abnormalities is primarily disseminated in journals specializing in AD, gerontology, and neurology (Figure 5). Among 333 journals, the top 10 accounted for 26.50% of publications. The Journal of the American Geriatrics Society ranked highest in IF (7.5) and citations (2,995), reflecting its authoritative role in this field (Table 2). Nevertheless, the average IF of prolific journals in this domain remains modest (3.4), indicating that while MCI gait research is widely published, achieving visibility in high-impact journals poses persistent challenges.

### 4.2 Research hotspots for gait studies on older adults with MCI

Research hotspots refer to scientific and academic problems that have garnered high attention from researchers during a specific period. In this study we explored the research hotspots in terms of active authors, co-occurring keywords, and co-cited reference.

#### 4.2.1 Analysis of the highly active authors

The most productive and popular co-cited author Verghese Joe is from Albert Einstein College of Medicine, the United States, and his research focus on disease and aging on gait and cognition in older adults. He also makes great influence of cognitively stimulating activities on reducing risk of dementia and mobility loss (Verghese et al., 1999), cognitive control and distribution of mobility (Verghese et al., 2002), and global health. His seminal contribution includes a cross-sectional study (Verghese et al., 2008) systematically compared gait characteristics across MCI subtypes and cognitively intact older adults. The study cohort comprised three distinct groups: 54 amnestic MCI (aMCI) patients, 62 non-amnestic MCI (naMCI) participants, and 295 age-matched controls with normal cognition. Multi-dimensional gait assessments demonstrated significant deterioration in both MCI subtypes relative to controls, with aMCI patients showing worse rhythm and variability, while naMCI participants exhibited marked reductions in velocity parameters compared to other groups. These findings not only established subtype-specific gait patterns strongly associated with functional decline but also suggested gait metrics as potential biomarkers for refining MCI diagnostic protocols and predicting dementia conversion.

Montero-odasso Manuel is from Western University, Canada, as the second rank of publications and citations. His research interests are in dementia and MCI treatment and prevention, as well as falls in the older adults. Especially, initiating dual-task (DT) gait to identify MCI. Besides, he chairs the World Fall Guidelines (Montero-Odasso et al., 2022), an initiative that develop and validate clinical practice guidelines for fall prevention and management. Since 2009, his team has published numerous studies on quantitative DT gait analysis in older adults with MCI (Montero-Odasso et al., 2009; Montero-Odasso et al., 2012a; Montero-Odasso et al., 2014; Montero-Odasso et al., 2017). His seminal work includes a prospective cohort study (Montero-Odasso et al., 2017) that conducted 6-year follow-up assessments of 112 community-dwelling older adults to investigate associations between DT gait performance and incident dementia in mild cognitive impairment patients. Results demonstrated no association between slow single-task gait speed (<0.8 m/s) and dementia progression, whereas elevated dual-task costs (DTC) while counting backward and naming animal showed significant associations with dementia conversion. Models remained robust after baseline cognitive adjustment, except for dichotomized gait DTC. These findings support shared pathogenic mechanisms underlying cognitive-motor dysfunction in MCI and propose DT gait abnormalities as potential motor biomarkers for dementia progression. (Montero-Odasso et al., 2017).

Tinetti Mary E. is from Yale University, the United States, possessing the strongest centrality in the field. Her current research focuses on clinical decision-making for older adults with multiple chronic conditions and the significance of universal health outcomes applicable across diseases in aging populations (Gill et al., 1995). She developed the *Tinetti Performance-Oriented Mobility Assessment (POMA)* (Tinetti, 1986) a tool widely employed to assess balance and gait in older adults and stroke survivors. The POMA emphasizes action-oriented rehabilitation evaluation, serving not only to measure mobility, balance, and fall risk but also to inform tailored fall prevention strategies, monitor longitudinal changes in mobility and balance, and evaluate rehabilitation efficacy (Tinetti, 1986).

#### 4.2.2 Analysis of the co-occurring keywords

Co-occurring keyword analysis revealed that gait research in MCI is predominantly applied to three major neurodegenerative diseases in older adults: dementia, AD, and Parkinson’s disease (PD).

Nearly all MCI cases ultimately progress to dementia, severely compromising activities of daily living (ADLs). Epidemiologically, AD constitutes the most prevalent dementia subtype, accounting for 50%–70% of cases (Rostagno, 2022). AD is characterized by progressive memory decline, often accompanied by impairments in speech, executive function, visuospatial skills, and behavioral abnormalities (Mantzavinos and Alexiou, 2017). MCI represents the early stage of AD’s disease continuum, where early diagnosis, intervention, and precision therapies critically delay progression (Rostagno, 2022). Based on MCI subtypes, aMCI typically advances to AD, whereas naMCI may evolve into vascular dementia or frontotemporal dementia (Bradfield, 2023). Notably, gait abnormalities manifest more prominently in aMCI than naMCI (Masse et al., 2021). Given the heterogeneity of dementia subtypes, MCI gait studies primarily target AD diagnosis and prevention.

Additionally, MCI gait research also informs early identification and intervention in PD. Prodromal Parkinson’s disease (pPD) describes the preclinical phase of neurodegeneration preceding motor symptoms (Aarsland et al., 2017). During pPD, non-motor or mild motor symptoms (e.g., cognitive impairment) emerge without meeting PD diagnostic criteria (Mahlknecht et al., 2022). Furthermore, MCI often marks the transition from pPD to PD and correlates with PD-dementia risk (Pan et al., 2022). A cohort study (Bougea et al., 2019) of 1,629 older adults (aged ≥65 years) demonstrated higher pPD prevalence in MCI patients than cognitively healthy controls, with pPD incidence linked to deficits across cognitive domains. Furthermore, the gradual decline of cognitive function (particularly in memory, execution, attention, and visuospatial function) drives gait abnormalities in PD.

#### 4.2.3 Analysis of the co-cited reference

Co-cited references denote pairs of publications cited simultaneously by one or more subsequent papers (Zhong et al., 2021). The greater the number of times two articles are cited by the same paper, the higher their similarity. Combined with the 5 most co-cited articles, we can see the applications of gait analysis on MCI is important, where gait analysis can be used to identify MCI, forecast AD progress, as a complementary approach to understanding brain impairment of MCI, evaluate fall risk and develop a motor-cognitive DT physical exercise protocol to intervene MCI early.

Among these, a systematic review from Bahureksa et al. (Bahureksa et al., 2017) had the highest frequency of co-citation. This article synthesized evidence on gait and balance parameters differentiating MCI patients from cognitively healthy older adults. The authors demonstrated that MCI disproportionately affects gait metrics under cognitive DT conditions and proposed motor assessments as diagnostic tools for early MCI detection and targeted intervention to delay dementia onset. A clinical study from Muir et al. (Muir et al., 2012), had the highest centrality of co-citation, focusing on gait assessment in older adults with MCI, AD and normal cognition under different DT challenges, stressing the advantages of DT gait analysis in identifying MCI and AD, highlighting the value of complex cognitive-motor integration tests in clinical assessment.

### 4.3 Global trends for gait studies on older adults with MCI

#### 4.3.1 Analysis the burst and timeline mapping of keywords

Keywords burst analysis and timeline mapping provide critical insights into evolving research trends and frontiers in gait studies on MCI. Over the past two decades, gait studies on MCI have evolved through distinct phases. Bibliometric indicators from the first decade reveal a focus on mechanistic investigations of gait performance in MCI (particularly under DT conditions), pathological correlates in specific cortical regions, and the neurodegenerative continuum linking MCI to AD and PD. Concurrently, significant attention was directed toward physical activity interventions in community-dwelling older adults. In the subsequent decade, research shifted toward leveraging wearable 3D gait analysis technologies to extract gait metrics and applying artificial intelligence (AI)-driven algorithms to develop diagnostic models for improved MCI detection accuracy.

A keyword-based cluster analysis delineated nine thematic clusters (Figure 7C), which were further examined via timeline mapping to identify emerging frontiers. The primary themes include: #0 physical activity, #1 gait, #2 intraindividual variability, #3 dementia, #4 brain atrophy, #5 postural control, #6 white matter hyperintensities, #7 hearing loss, #8 vascular dementia. Among these themes, cluster (#0) exhibited the highest keyword density, strongest intra-cluster cohesion, and the longest temporal span, underscoring its sustained dominance as a foundational and evolving research priority in this field.

Keywords exhibiting strong citation bursts are indicative of emerging research frontiers (Figure 7B). Terms such as “validation,” “reliability,” “diagnosis,” and “intervention” are projected to dominate future studies, signaling three pivotal research directions in gait studies on MCI:

(1) Validation and Reliability of gait Assessment: Validation and reliability reflect the precision and consistency of measurement methodologies. Current 3D gait analysis systems employ marker-based motion-capture technology to objectively quantify multi-dimensional gait parameters (Lo et al., 2022; Martini et al., 2022). The platform achieves sub-millimeter accuracy, positioning them as robust tools for MCI identification (Scataglini et al., 2024). Systematic characterization of gait patterns and analysis of gait abnormality etiologies have identified subtle biomechanical signatures linked to MCI (Marquis et al., 2002). While spatiotemporal parameters (e.g., gait speed, stride length, stance/swing phase duration) often overlap between MCI and cognitively healthy older adults, their dynamic alterations serve as critical biomarkers for MCI progression (Guan et al., 2022; Lindh-Rengifo et al., 2022; Windham et al., 2022; Mo et al., 2024). Joint angle metrics, particularly toe-off and heel-contact angles, further discriminate cognitive impairment levels (Ni et al., 2021; Wang et al., 2023).

Additionally, DT gait analysis add additional cognitive load (such as cognitive tasks involving attention, memory, execution ability, etc.) to the walking task so that to expose functional deficits (McIsaac et al., 2015). Underlying the DT gait paradigm, MCI patients with insufficient cognitive reserve are difficult to complete the walking and cognitive tasks simultaneously, resulting in obvious gait changes (e.g., reduced gait speed), which enhance diagnostic sensitivity of MCI (Yang et al., 2020; Weng et al., 2023). Such paradigms also predict fall risk and evaluate cognitive-motor integration, demonstrating utility in MCI screening (Ghai et al., 2017; Liu et al., 2017).

However, the absence of standardized protocols for DT paradigm design limits comparability across studies. Motor-cognitive tasks (e.g., walking + counting backward) and dual-motor tasks (e.g., walking + carrying objects) engage distinct cognitive domains, yielding variable diagnostic efficacy (Kelly et al., 2010). Validated paradigms combining walking with secondary tasks like serial subtraction, word recall, or obstacle negotiation show promise in MCI identification (Bahureksa et al., 2017). Hunter et al. (Hunter et al., 2018) established a difficulty hierarchy for DT paradigms, revealing that motor-cognitive tasks (e.g., walking + counting backward from 100) elicit greater gait deficits in MCI patients than dual-motor tasks, underscoring the need for task complexity to optimize diagnostic validity Similarly, Ghoraani et al. (2021) advocate for cognitively demanding DT paradigms to enhance MCI differentiation from healthy controls.

(2) Diagnosis challenges and innovations: The clinical diagnosis of MCI remains challenging due to the absence of standardized diagnostic criteria. First, neuropathological studies have confirmed that abnormal deposition of amyloid-β (Aβ) protein is closely associated with memory and language cognitive domains, and its dysregulated production and clearance mechanisms have been identified as potential initiating factors for AD (Harrington et al., 2013), providing critical insights for MCI diagnosis. Tau positron emission tomography (Tau-PET) demonstrates promise as a biomarker for predicting cognitive trajectory in preclinical and prodromal AD stages (Ossenkoppele et al., 2021). However, the lack of definitive MCI-specific biomarkers persists, as its pathological features substantially overlap with dementia and age-related cognitive changes (Petersen et al., 2014). Neuropathological and longitudinal studies highlight the heterogeneity of MCI, with pathological markers existing along a spectrum between normal cognition and dementia (Stephan et al., 2012). Furthermore, MCI’s multifactorial etiology including spanning vascular, neurodegenerative, and metabolic pathways, complicates the identification of a singular diagnostic biomarker. Besides, current diagnostic practices rely on semi-quantitative neuropsychological tests such as the Mini-Mental State Examination (MMSE) (Liu et al., 2021), Montreal Cognitive Assessment (MoCA) (Nasreddine et al., 2005), Clinical Dementia Rating (CDR) (Tariot et al., 2024). However, these tools exhibit limited sensitivity, high subjectivity, and cultural bias. Consequently, establishing a comprehensive, multidimensional diagnostic framework has emerged as an urgent clinical imperative.

Gait analysis has become an effective tool to assist in the diagnosis of MCI, yet traditional gait and balance tests cannot detect subtle motor impairments associated with MCI. DT-based gait analysis has demonstrated significant potential, revealing gait parameters closely linked to MCI (such as stride length variability, swing phase asymmetry) (Bahureksa et al., 2017). However, these parameters are susceptible to intraindividual variability, environmental confounders, and multicollinearity, limiting their standalone diagnostic utility (Verghese et al., 2008). Machine learning (ML) as a AI-driven algorithms can address these limitations by identifying and integrating high-impact gait features (e.g., dynamic joint angles, DTC) into predictive models, thereby enhancing diagnostic accuracy (Tao. et al., 2022). For instance, Ghoraani et al. (2021) developed a Support Vector Machine (SVM)-based DT gait assessment method to objectively diagnose MCI and AD, and effectively distinguish them from cognitively intact subjects. Despite these advances, optimal ML architectures (e.g., deep learning vs ensemble methods) for MCI diagnosis remain underexplored, necessitating prospective multicenter studies to validate digital biomarker-driven diagnostic frameworks.

(3) Intervention strategies: Regarding infusion therapies aimed at delaying the progression of MCI, current efforts primarily focus on clearing pathological proteins Aβ and tau. Significant breakthroughs have been made in Aβ-targeted disease-modifying therapies (DMT), with multiple anti-Aβ monoclonal antibodies completing Phase III clinical trials (Leuzy et al., 2023). Among these, lecanemab has received marketing approval from the U.S. Food and Drug Administration (FDA), Japan Pharmaceuticals and Medical Devices Agency (PMDA), and China National Medical Products Administration (NMPA) for treating MCI patients (Tahami Monfared et al., 2023). In addition to DMT drugs, symptomatic medications may be used in clinical management to enhance cognition or alleviate neuropsychiatric symptoms in MCI patients. However, although cholinesterase inhibitors are the primary symptomatic treatment for AD dementia, their efficacy in MCI remains uncertain (Han et al., 2019).

Current non-pharmacological approaches for MCI treatment are also gaining attention. Multimodal interventions targeting modifiable risk factors such as nutrition, sleep optimization, physical activity, and social engagement may help delay cognitive decline (Kivipelto et al., 2013). Among these, cognitive-motor DT training has emerged as the most widely validated intervention. Integrated DT protocols synergistically enhance cognitive function and ADLs in MCI patients, outperforming single-domain interventions (Gavelin et al., 2021). A systematic review by Ali et al. (Ali et al., 2022) demonstrated that DT training significantly improves cognitive performance, gait stability, and balance in older adults across cognitive impairment stages, though optimal intensity and duration require further standardization.

These trends highlight three priorities for future research: (1) establishing validated DT gait paradigms for early MCI detection, (2) leveraging AI-driven algorithm to develop gait-based diagnostic models, and (3) personalizing cognitive-motor DT interventions to mitigate fall risk, enhance functional independence, and decelerate dementia progression.

#### 4.3.2 Analysis the burst and timeline mapping of references

In terms to the timeline graph of references (Figure 8C), Cluster (#0 exercise) stands out with the highest number of articles (121) and exhibits robust bursts. Exercise is a cornerstone intervention for promoting healthy aging and mitigating MCI. However, optimal exercise modalities for MCI remain debated. Nuzum et al. (Nuzum et al., 2020) demonstrated that aerobic exercise enhances cognitive performance and functional independence in older adults with cognitive decline (including MCI and dementia). Conversely, Huang et al. (Huang et al., 2022) synthesized evidence favoring multicomponent exercise (such as integrating cognitive-motor DT training), as superior for preserving global cognition and executive function in MCI.

Through comprehensive analysis, Cluster (#4 fall prevention) represents a research priority in recent years, driven by the high prevalence of postural instability and falls in MCI populations Attentional and executive dysfunction in MCI impairs movement regulation, elevating fall risk during daily activities (Del Din et al., 2020). Neuroimaging studies by Holtzer et al. (Holtzer and Izzetoglu, 2020) revealed diminished prefrontal cortex (PFC) activation during DT walking in MCI patients, linking reduced PFC efficiency to impaired executive control and gait instability. Such findings underscore the need for mechanistic research to inform fall prevention strategies.

By comparison, Cluster (#12 wearable technology) has surged as an emerging frontier over the past 3 years. Wearables enable high-resolution gait analysis through ambulatory motion capture (Kuan et al., 2021) and underpin virtual reality (VR)-based rehabilitation systems (Saab et al., 2021). With most MCI patients residing in community settings, demand for home-based wearable devices and VR platforms is growing (Liao et al., 2020). Tortora et al. (Tortora et al., 2024)highlighted VR’s ecological validity and adaptability for cognitive-motor training, demonstrating efficacy comparable to conventional therapies. However, limited clinical validation and standardized protocols necessitate further research to optimize wearable-driven interventions for MCI populations.

Recent references with significant citation bursts may reveal emerging trends in global research. Notable examples include the following (Figure 8B): Petersen et al. (2018) emphasized the importance of modifiable risk factors such as walking ability in MCI identification and intervention, which will be the continuous focus of researchers in the future. The Asian Working Group for Sarcopenia (AWGS), led by Chen et al. updated on sarcopenia diagnosis and treatment in 2019 (Chen et al., 2020). The AWGS focused on the association between MCI and sarcopenia, with slow gait speed as an important gait feature, and found that exercise not only improved muscle strength but also improved cognitive ability. Grande et al. (2019) conducted a scoping review of 39 studies examining the association between gait speed and cognitive outcomes. Their findings support the use of gait speed as a predictor of cognitive decline and dementia in cognitively intact older adults and those with initial cognitive impairment.

### 4.4 Limitation

Although this bibliometric analysis provides valuable insights into MCI gait research, several limitations must be acknowledged. First, the study relied exclusively on the WoSCC database due to technical constraints and the scope of the visualization software (including CiteSpace and VOSviewer), potentially omitting relevant literature from other databases. While, other databases were consulted as supplementary references to identify pertinent publications and examine emerging trends and research frontiers in the field. Future research will not only consider using other format conversion tools to export data from databases such as PubMed into WoS-style formatting, but also incorporate cited and citing reference information from citation databases like EI Compendex and Scopus. This approach would expand the literature data volume to enable more comprehensive and detailed visual network analysis of this field. Second, excluding non-English articles may have introduced selection bias. Third, bibliometric methods inherently lack the capacity to evaluate the scientific validity or methodological rigor of publications. For example, recent studies may be underrepresented due to delayed citation accrual, and citation metrics can be skewed by temporal biases. Consequently, certain articles may have been overlooked, underscoring the need for methodological refinements in future bibliometric investigations of MCI-related gait research.

## 5 Conclusion

The application of gait analysis in detecting subtle gait changes associated with MCI holds significant research potential, particularly for aiding diagnosis, preventing disease progression in dementia, AD, and PD, exploring links between cognitive and motor decline in older adults, and evaluating multi-component exercise interventions. CiteSpace and VOSviewer visual analyses indicate sustained prominence in MCI gait research over the past 5 years. Historically, the United States has dominated this field globally, though collaboration among other countries and institutions remains fragmented, as many entered the field only recently. Leading journals and authors predominantly focus on AD, gerontology, and neurology. Keyword and reference analyses suggest that DT gait analysis, a critical tool for identifying MCI-specific gait patterns, will see expanded use in future diagnostic research. Furthermore, early diagnosis and interventions for dementia, AD, and PD—three degenerative diseases closely tied to MCI progression—are anticipated to gain traction. These areas are likely to remain focal points in MCI gait studies. Concurrently, advancements in the validity and reliability of gait analysis are accelerating, with DT walking paradigms demonstrating notable utility and AI-driven diagnostic models enabling multidimensional gait parameter analysis. However, optimal task paradigms and diagnostic frameworks remain underexplored due to limited research. While exercise is widely recognized for improving cognitive and motor function in MCI, inconsistent evaluation criteria necessitate further multi-center studies to identify optimal physical activity regimens for MCI patients. In summary, this analysis fosters academic collaboration and provides a foundation for identifying research hotspots and emerging trends in MCI-related gait studies.

## Data Availability

The original contributions presented in the study are included in the article/supplementary material, further inquiries can be directed to the corresponding author.
